# Anaphylactoid Reactions Following Red Blood Cell Transfusion in a Patient with Previously Undiagnosed Immunoglobulin A Deficiency: Case Report

**DOI:** 10.5152/TJAR.2023.22133

**Published:** 2023-04-01

**Authors:** Hande Güngör, Tümay Uludağ Yanaral, Emine Uzunoğlu, Pelin Karaaslan

**Affiliations:** 1 Department of Anaesthesiology and Reanimation, İstanbul Medipol University School of Medicine, İstanbul, Turkey; 2 Department of Anaesthesiology and Reanimation, İstanbul Medipol University International School of Medicine, İstanbul, Turkey

**Keywords:** Anaphylaxis, blood products, immunoglobulin A, perioperative care, transfusion

## Abstract

A 73-year-old, 104 kg female patient was hospitalised for debulking and low anterior colon resection operations. Anaphylactoid symptoms developed when administering erythrocyte suspension and fresh frozen plasma. Through the immediate haematology department consultation, it was found that the patient might possibly have immunoglobulin A deficiency. Immunoglobulin A level was found to be very low in the patient’s blood sample, which was sent intraoperatively to verify the diagnosis. This case report discusses a sudden anaphylactic reaction that occurred as a result of a blood transfusion in a previously undiagnosed immunoglobulin A deficiency.

**Main Points**

- Anesthetic management in IgA deficiency is challenging.
- Anaphylactoid reactions following blood transfusion in individuals with IgA deficiency are rare but serious events that require prompt recognition, appropriate management and trated quickly.
- During the transfusion, it is important that the patient is monitored for any adverse events by the medical staff.
- Close collaboration between the patient, anesthetist, and blood bank can help to prevent future reactions and ensure the safe use of blood products.

## Introduction

Immunoglobulin A (IgA) is the second most common antibody found in human serum. It’s secretory form can inactivate viruses, toxins, and bacteria; bind to toxins; and prevent bacteria from adhering to mucosal epithelial cells.^1,2^ Selective IgA deficiency, which is the most common primary immunodeficiency disease (incidence up to 1/400), is a temporary or permanent disorder characterised by the lack of secretory IgA and a serum IgA level below the normal range, in the setting of normal serum IgM and serum IgG levels.^2^ Majority of patients with IgA deficiency are asymptomatic.^2,3^ In asymptomatic patients, severe recurrent sinopulmonary infections, autoimmune diseases, allergic disorders, malignancies, chromosomal and cytogenetic problems, and allergic reactions to blood products may occur.^4^

In the subset of IgA-deficient patients in whom anti-IgA antibodies are formed, anaphylactic reaction to blood products may occur. The incidence of anti-IgA-mediated anaphylactic transfusion reactions is very low. Eighty-six out of 2.5 million blood transfusions in the UK in 2015 were reported as anaphylactoid and only 1 of them was anti-IgA mediated.^5^

This case report discusses a sudden anaphylactic reaction that occurred as a result of a blood transfusion in a previously undiagnosed patient. Written consent has been obtained from the patient’s daughter indicating her approval for publication.

## Case Presentation

A 73-year-old, 104 kg female patient arrived at our hospital with complaints of abdominal pain and swelling in the abdomen. The patient was diagnosed with colon-invasive ovarian cancer upon completion of examination, evaluation, and imaging tests. The patient was subsequently hospitalised for debulking and low anterior colon resection operations planned to be performed by the gynaecology and general surgery departments.

The patient was evaluated preoperatively by the anaesthesiologist and did not have any comorbidities or active complaints aside from hypertension and obesity. The only medication she received before the presentation was aspirin and an antihypertensive drug. She had no history of smoking, alcohol, substance abuse, or known allergies. Electrocardiography (ECG) and echocardiography (ECHO) were performed during the cardiology consultation for the patient, although she did not have any active cardiac complaints. The ECG revealed a normal sinus rhythm and an incomplete right bundle branch block. According to the ECHO report, the patient had an ejection fraction of 65%, mild aortic regurgitation, mild mitral regurgitation, and moderate tricuspid regurgitation. The patient was considered as operable with moderate cardiac risk. The laboratory findings of the patient were as follows: haemoglobin (Hgb) was 11.5 g dL-1, haematocrit was 33.2, platelet was 245 000, and international normalized ratio (INR) was 1.07. In case of bleeding during the procedure, 5 units of erythrocyte suspension (ES) and 5 units of fresh frozen plasma (FFP) were prepared by the blood transfusion centre.

Since the patient’s Hgb level reached 8.5 g dL^-1^ in blood gas analysis and clinically suspected active bleeding was present, it was decided to perform ES replacement as intraoperative bleeding was obvious. A possible allergic reaction to blood transfusion was considered as the patient experienced hypotension (BP: 85/40 mmHg), tachycardia (120 beats min^-1^), a decrease in end-tidal CO_2_ (27 mmHg), and an increase in airway pressure (peak inspiratory pressure: 41 cm H_2_O) following nearly 100 mL of ES transfusion. Transfusion was subsequently ceased. The suitability of the blood bag and blood to the patient was checked once more. The blood bag was then sent to the transfusion unit for further examination. The patient was administered 250 mg of methylprednisolone, 45.5 mg of pheniramine hydrogen maleate, and 0.01 mg of adrenaline. Crystalloid volume loading was performed to compensate for the volume deficit in the patient. As the hypotension continued, a low-dose noradrenaline infusion (0.1 µg kg^-1^ min^-1^) was started, and the operation continued. Upon the same reaction, symptoms developed when administering FFP given for volume replacement, and the haematology department was consulted intraoperatively. The haematology department considered that the patient might possibly have IgA deficiency. A blood sample taken from the patient was sent to the laboratory to confirm the diagnosis. As it was impossible to perform blood and blood product transfusion on the patient, the surgical team was warned to end the operation as soon as possible. It was decided that all blood products should be administered to the patient after washing. However, the transfusion centre informed that washing the blood and blood products would last for at least 3 hours, and they began working immediately. The perioperative replacement requirement was met with crystalloid, colloid, and human albumin. Immunoglobulin A level was found to be 12.6 mg dL^-1^ (ref: 70-400 mg dL^-1^) in the patient’s blood sample, which was sent intraoperatively. Her diagnosis was confirmed to be selective IgA deficiency. The patient was transferred to the intensive care unit (ICU) under noradrenaline infusion with orotracheal intubation for postoperative follow-up and treatment. The patient’s relatives were interviewed regarding details of her past medical history after the ICU transfer. It was found that the patient had a previous history of gastrointestinal bleeding, and an allergy developed when attempts were made to administer blood products after this bleeding. At that time, they were told that it was considered to be a local allergic or transfusion reaction.

Throughout the follow-ups in the ICU, the Hgb level decreased to 3.6 g dL^-1^. The patient received 6 units of washed ES replacement. No problems were encountered in the transfusion of washed blood products. Upon improvement of the patient’s haemodynamics, the noradrenaline infusion was ceased, and the patient was awakened. The Haematology department was consulted for the patient with the follow-up values of Hgb level at 11.6 g dL^-1^; fibrinogen at 40, and INR at 6.06. They offered Eptacog alfa-recombinant factor 7 A (rFVIIa) 2 mg treatment. The patient’s general condition worsened throughout the follow-up due to oozing-type bleeding simulating disseminated intravascular coagulation diagnosis. Her blood pressure and cardiac output failed to improve even though inotropic and vasoconstrictor infusions were restarted. She was unfortunately accepted as exitus on the third day of her ICU follow-up due to multiorgan failure.

## Discussion

There are several suggested mechanisms of how IgG anti-IgA antibodies can cause anaphylactic transfusion reactions. Some reports suggest that IgG anti-IgA antibodies may be a biomarker that increases the risk of non-IgE-mediated anaphylaxis. Another mechanism might be IgG anti-IgA antibodies reacting with infused IgA; therefore, blood products with higher levels of IgA would be more likely to cause a reaction.^6^ Unfortunately, the exact mechanism of how IgG anti-IgA antibodies cause anaphylactic transfusion reactions needs further investigation.

Anti-IgA-induced anaphylactic transfusion reactions are extremely rare. Since there is a risk of developing a reaction against blood products in patients with known IgA deficiency, IgA-poor or washed ES should be prepared if blood replacement is to be performed in these patients. Washed red blood cells and plasma or platelets from known IgA-deficient donors is the safest method since anti-IgA antibodies could not find and fight any IgA body. Patients should be informed about the potential anaphylactic reaction that may occur following the transfusion of blood products. It is recommended that these patients wear a medical alert bracelet. In our patient, such preparation could not be made since the patient did not know during the anaesthesia evaluation that she had IgA deficiency. The history of previous blood transfusion and allergic reactions should be questioned in detail in all previously suspected transfusion patients. All blood products should be administered carefully in patients with a known diagnosis, and all members of the team should be prepared for possible anaphylaxis. In the literature, cases are usually from the ones with a known diagnosis of IgA deficiency in whom serum IgA levels were low. These anaphylactic reactions might be induced by the presence of anti-IgA antibody, since the level of antibody titres in their serum is elevated.^7^ Sudden cardiac arrest, shock, hypotension, angioedema, urticaria, stridor, and wheezing may occur in these patients. In patients where anaphylaxis does occur, emergency intervention is important. Immediately, the transfusion should be stopped, and epinephrine is to be administered. Hypotensive patients should be adequately resuscitated with intravenous fluids. All the steps were performed definitely in this order in our patient too. There have been cases in which rFVIIa has been used in systemic bleeding disorders. Fortunately, rFVIIa was present in our hospital and we were able to transfuse safely since it does not contain IgA.^8,9^

Due to the heterogeneity of IgA deficiency and the lack of a specific treatment, each patient should be treated with an individualised approach. Recommendations for this include periodic monitoring, prophylaxis, and treatment of infections with antibiotics, treatment of associated allergic and autoimmune conditions, replacement therapy with intravenous or subcutaneous immunoglobulins, administration of polyvalent pneumococcal vaccines, and patient education.^10–12^

## Conclusion

Anaesthesiologists do not routinely ask for serum IgA levels, in particular when preparing patients for operations that may require a blood transfusion. Hence, the medical history of the patient should be taken meticulously before the operation in patients who will probably receive blood transfusion intraoperatively, and the history of previous blood transfusion and allergic reactions should be questioned in detail. Even though it was asked to our patient, she probably could not remember the point. If known, the procedure should be postponed for further examination and evaluation in patients with suspected allergic reactions. In patients with a history of a transfusion reaction, the blood sample should be sent for the analysis of serum IgA level, and IgA-poor or washed ES should be preferred during transfusion. As this process takes a long time, the blood transfusion centre should be contacted and blood products should be prepared before the operation. Blood products should be provided to these patients with caution, and the whole team should be prepared for possible anaphylaxis.
